# Role of Oral Microbiota in Cancer Development

**DOI:** 10.3390/microorganisms7010020

**Published:** 2019-01-13

**Authors:** Tomasz M. Karpiński

**Affiliations:** Department of Medical Microbiology, Poznań University of Medical Sciences, Wieniawskiego 3, 61-712 Poznań, Poland; tkarpin@ump.edu.pl or tkarpin@interia.pl; Tel.: +48-61-854-61-38

**Keywords:** oral microbiota, oral cancer, colorectal cancer, *Porphyromonas gingivalis*, *Fusobacterium nucleatum*, *Streptococcus* sp., chronic inflammation, antiapoptotic activity, cancerogenic substances

## Abstract

Nowadays cancer is the second main cause of death in the world. The most known bacterial carcinogen is *Helicobacter pylori*. Pathogens that can have an impact on cancer development in the gastrointestinal tract are also found in the oral cavity. Some specific species have been identified that correlate strongly with oral cancer, such as *Streptococcus* sp., *Peptostreptococcus* sp., *Prevotella* sp., *Fusobacterium* sp., *Porphyromonas gingivalis*, and *Capnocytophaga gingivalis*. Many works have also shown that the oral periopathogens *Fusobacterium nucleatum* and *Porphyromonas gingivalis* play an important role in the development of colorectal and pancreatic cancer. Three mechanisms of action have been suggested in regard to the role of oral microbiota in the pathogenesis of cancer. The first is bacterial stimulation of chronic inflammation. Inflammatory mediators produced in this process cause or facilitate cell proliferation, mutagenesis, oncogene activation, and angiogenesis. The second mechanism attributed to bacteria that may influence the pathogenesis of cancers by affecting cell proliferation is the activation of NF-κB and inhibition of cellular apoptosis. In the third mechanism, bacteria produce some substances that act in a carcinogenic manner. This review presents potentially oncogenic oral bacteria and possible mechanisms of their action on the carcinogenesis of human cells.

## 1. Introduction

Nowadays cancer is the second main cause of death in the world. It is estimated that in 2018 about 9.6 million people will have died from cancer. Among men, the most common types of cancers are lung, prostate, colorectal, and stomach cancers, while among women the most common are breast, colorectal, lung, and cervix cancers [1]. Cancer of the oral cavity is one of the most common malignancies [2]. According to the World Health Organization (WHO), there are an estimated 657,000 new cases of cancers of the oral cavity and pharynx each year, and more than 330,000 deaths [3]. Oral squamous cell carcinomas (OSCCs) constitute more than 90% of oral and oropharyngeal cancer and the main etiological factor is the synergistic effect of tobacco and alcohol use [4].

The most well-known bacterium associated with the development of cancer in humans is *Helicobacter pylori*, which is defined as a class I carcinogen [5]. *H. pylori* is an etiological agent of peptic ulcer disease, chronic gastritis, gastric adenocarcinoma, and gastric mucosa-associated lymphoid tissue (MALT) lymphoma, with intestinal metaplasia [6]. At the same time, there are data suggesting the role of oral cavity bacteria in the development of cancer. Such bacteria can be responsible for oral cancers, as well as tumors of the gastrointestinal tract. Some specific bacteria have been identified that correlate strongly with OSCCs, such as *Streptococcus* sp., *Peptostreptococcus* sp., *Prevotella* sp., *Porphyromonas gingivalis*, and *Capnocytophaga gingivalis* [7,8,9,10,11,12,13]. Oral cancer and epithelial precursor lesions are also linked with bacteria from genera *Fusobacterium*, *Veillonella*, *Actinomyces*, *Clostridium*, *Haemophilus*, and Enterobacteriaceae [14]. Many works have also shown that oral pathogens are essential in the development of colorectal and pancreatic cancer. Two periopathogenic species in particular have been frequently mentioned: *Fusobacterium nucleatum* and *Porphyromonas gingivalis* [15,16,17,18,19,20,21].

Literature was searched from articles published in PubMed/MEDLINE between 2000 and 2018 using combinations of the following keywords: “bacteria”, “microbiome”, “oral”, “oral cavity”, “cancer”, “carcinogenesis”, “inflammation”, “cytokine”, “apoptosis”, and “carcinogen”. Titles and abstracts of found papers were examined to order to determine which articles to exclude or include in the review. From the references of included articles, additional works were selected. Finally, ninety-eight articles were included in this narrative review.

In this review, potentially oncogenic oral bacteria are presented along with the possible mechanisms of their action on carcinogenesis of human cells.

## 2. Potentially Oncogenic Oral Bacteria

Mager et al. tested 40 bacterial oral species from a group of cancer-free individuals and from a group of subjects with oral squamous cell carcinoma (OSCC). The levels of three species (*Capnocytophaga gingivalis*, *Prevotella melaninogenica*, and *Streptococcus mitis*) were elevated in the saliva of patients suffering from OSCC. These three bacterial species were suggested as diagnostic markers and were found to predict 80% of cancer cases [8]. Studies by Nagy et al. have shown a higher number of oral bacteria associated with keratinizing squamous cell carcinomas of the following species: *Veillonella* sp., *Fusobacterium* sp., *Prevotella* sp., *Porphyromonas* sp., *Actinomyces* sp., *Clostridium* sp., *Haemophilus* sp., *Streptococcus* spp., and Enterobacteriaceae [14].

Among streptococci, *Streptococcus anginosus* seems to be an especially relevant marker of head, neck, and esophageal cancers [7,22,23]. In studies of Sakamoto et al., oral streptococci (*Streptococcus intermedius*, *S. constellatus*, *S. oralis*, *S. mitis*, *S. sanguis*, *S. salivarius*) were the most common isolates from cervical lymph nodes in patients with oral cancer. Among the anaerobic bacteria, *Peptostreptococcus* spp. dominated [24]. Some papers have reported that other genera are linked with OSCCs. Lee et al. revealed significant differences between epithelial precursor lesion and cancer patients in five genera: *Bacillus* sp., *Enterococcus* sp., *Parvimonas* sp., *Peptostreptococcus* sp., and *Slackia* sp. [13], whereas Pushalkar et al. highly associated OSCC tumor sites with the following species: *Streptococcus* sp., *Peptostreptococcus stomatis*, *Gemella* sp., and *Johnsonella ignava* [10]. 

Taking the above into consideration, the most often observed oral bacteria in OSCCs are *Streptococcus* sp., *Peptostreptococcus* sp., *Prevotella* sp., *Porphyromonas gingivalis*, and *Capnocytophaga gingivalis* [7,8,9,10,11,12,13].

Oral bacteria are also detected in tumors outside the oral cavity and appear in patients with colorectal and pancreatic cancers. In cases of colorectal cancer, two species are especially prominent: *Fusobacterium nucleatum* and *Porphyromonas gingivalis* [15,16,17,19,25,26]. A high abundance of *Fusobacterium* (in particular *F. nucleatum*) at colorectal cancer sites has been associated with regional lymph node metastases [15] and tumor location (2% in rectum and approx. 11% in cecum) [27]. In pancreatic cancers, in addition to *Fusobacterium nucleatum* and *Porphyromonas gingivalis,* strains of *Aggregatibacter actinomycetemcomitans*, *Neisseria elongata*, and *Streptococcus mitis* have been described [18,20,21,28]. Oral bacteria from genera *Capnocytophaga* and *Veillonella* are reportedly present in increased amounts in lung cancer patients [29].

Table 1 presents oral bacteria that are associated with specific cancer types.

## 3. Mechanisms of Carcinogenic Action of Oral Bacteria

**Figure 1:**
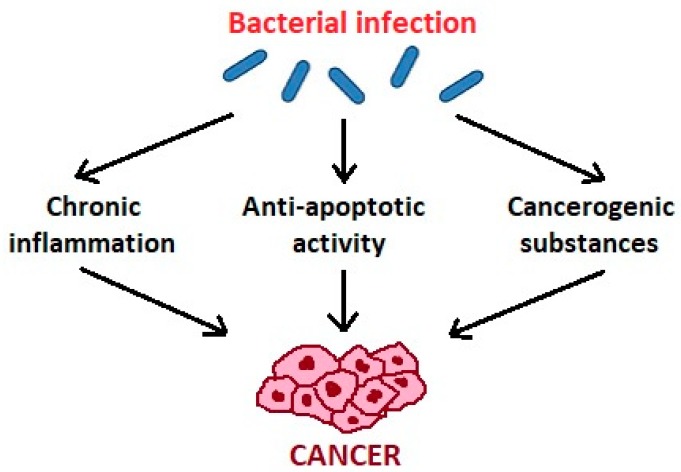
The influence of oral bacteria in the pathogenesis of cancer.

Zhang et al. [34] suggest three mechanisms of action of oral microbiota in the pathogenesis of cancer (Figure 1). The first is bacterial stimulation of chronic inflammation. Inflammatory mediators produced in this process cause or facilitate cell proliferation, mutagenesis, oncogene activation, and angiogenesis. Regarding the second mechanism, bacteria may influence the pathogenesis of cancers by affecting cell proliferation, cytoskeletal rearrangements, activation of NF-κB, and inhibition of cellular apoptosis. As for the third mechanism, bacteria produce some substances that may be carcinogenic [34].

### 3.1. Chronic Inflammatory Process

Oral bacteria, especially anaerobic species such as *Porphyromonas*, *Prevotella*, and *Fusobacterium*, are responsible for periodontal diseases and lead to chronic inflammatory processes. These bacteria stimulate production of inflammatory mediators and have harmful effects on fibroblasts, epithelial and endothelial cells, and extracellular matrix components. Periodontal pathogens affect growth of local concentrations of various cytokines including interleukin-1β (IL-1β), IL-6, IL-17, IL-23, tumor necrosis factor-α (TNF-α), and matrix metalloproteinases MMP-8 and MMP-9 [35].

In tissues of the periodontium, monocytes/macrophages, neutrophils, fibroblasts, and mast cells are the primary sources of IL-1β. Among others, these cells synthesize IL-1β in response to activation from the influence of lipopolysaccharide (LPS), the main component of Gram-negative bacteria cell walls. IL-1β causes osteoclast formation and bone resorption, which leads to local inflammatory changes in the periodontium. Moreover, this cytokine stimulates the release of phospholipase A2, prostaglandins (PG), acute phase proteins, as well as proinflammatory cytokine IL-6, tumor necrosis factor (TNF), and many metalloproteinases (MMPs) [36,37]. IL-1β activates endothelial cells to produce vascular endothelial growth factor (VEGF) and other proangiogenic factors (e.g., TNF) which provide an inflammatory microenvironment for angiogenesis and tumor progression [38]. High IL-1β content is associated with tumor invasiveness, migration, and more aggressive tumor phenotype [39,40]. In the study by Wang et al., IL-1β was linked to lower expression of E-cadherin, which promotes cell migration [41]. Low E-cadherin expression is correlated with disorders of cellular functions, growth inhibition, apoptosis, cell cycle arrest, and differentiation. It leads to aggressive carcinoma, higher invasiveness, and low patient survival [42,43]. Simultaneously, IL-1β induces MMP-9, which has a role in local extracellular matrix degradation and tumor invasion. The loss of E-cadherin-mediated adhesion and increase of MMP-9-induced migration are important markers of the transition of epithelial tumors from a benign to an invasive state [41]. 

Another important pro-inflammatory cytokine is IL-6. It is produced by many cells of periodontal tissues in response to stimulation under the influence of LPS and proinflammatory cytokines IL-1β and TNF. IL-6 induces bone resorption and stimulates synthesis of acute phase proteins, chemokines, and PGE2 [44,45]. IL-6 induces oxidative stress and can lead to a transient accumulation of H_2_O_2_ in mitochondria and consequently to mitochondrial damage [46,47]. IL-6 also affects the process of invasion and metastasis by increasing the expression of matrix metalloproteinases (MMPs) [48]. Additionally, this cytokine upregulates the expression of various adhesion molecules (ICAMs) and endothelial leukocyte adhesion molecules (ELAMs), which cause adhesion of tumor cells to endothelial cells, and therefore have an impact on tumors spreading [49]. Most genes that are targeted by IL-6 are involved in cell cycle progression and suppression of apoptosis. By influencing anti-apoptotic pathways, IL-6 may have an impact on cancer development [50].

Also, one of the major cytokines of the inflammatory response is TNF-α. This cytokine is synthesized among others by monocytes/macrophages, neutrophils, fibroblasts, lymphocytes, and mast cells. This cytokine is secreted in response to many factors, including bacterial LPS. TNF-α strongly induces the production of reactive oxygen compounds, leukotrienes, prostaglandins, and metalloproteinases [51]. TNF leads to a reduction in the number of osteogenic cells and fibroblasts [52]. In contrast to high doses of TNF-α, which are related to tumor destruction, exposure to low doses of this molecule are related to tumor promotion [53]. Activation of oncogenic signaling pathways in epithelial cells, including Wnt and NF-κB, is critical for TNF-α-induced tumor growth [54]. Also, TNF-α possesses the ability to induce DNA damage by production of reactive oxygen species [55]. TNF-α has been shown to influence processes of motility and invasion by induction of MMPs expression [56] and simulation of the production of various angiogenic factors, such as interleukin-8, VEGF, and basic fibroblast growth factor [57].

### 3.2. Antiapoptotic Activity

**Figure 2:**
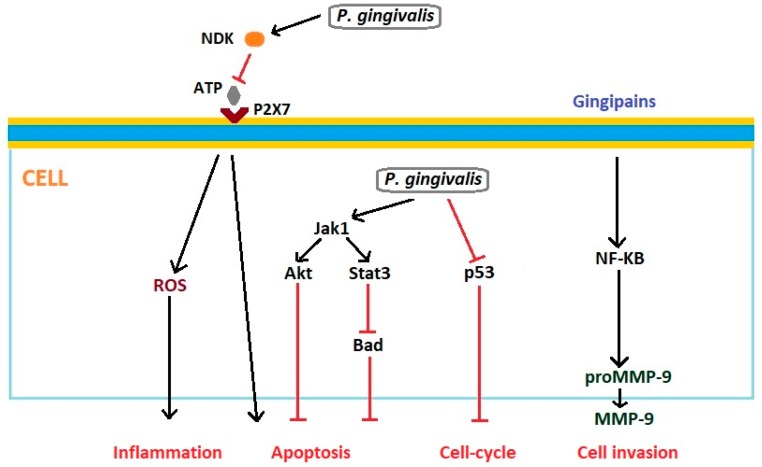
Interactions between *Porphyromonas gingivalis* and epithelial cells that can affect development of oncogenic phenotype. Based on Gholizadeh et al. [58] and Whitmore and Lamont [61]. Akt: protein kinase B; ATP: Adenosine triphosphate; Bad: Bcl-2-associated death promoter; Jak1: Janus kinase 1; MMP: metalloproteinase; NDK: nucleoside diphosphate kinase; NF-kB: nuclear factor kappa B; P2X7: Purinergic receptor; p53: Tumor protein p53; ROS: reactive oxygen species; Stat3: Signal transducer and activator of transcription 3.

*Porphyromonas gingivalis* acts antiapoptotically by modulation of several pathways [58]. Intracellular *P. gingivalis* activates antiapoptotic Jak1/Akt/Stat3 signaling, which controls intrinsic mitochondrial apoptosis pathways [59,60]. This pathogen also accelerates progression through the S-phase of the cell cycle by manipulation of cyclin/CDK (cyclin-dependent kinase) activity and reduces the level of the p53 tumor suppressor [61]. *P. gingivalis* causes significant phosphorylation of pro-apoptotic Bad at the mitochondrial membrane, and its inhibition, with enhancement of the ratio of Bcl2 (anti-apoptotic) and Bax (pro-apoptotic). *P. gingivalis* inactivates pro-apoptotic Bad through Akt and simultaneously inhibits caspase-9 independently of Akt [62]. Nakhjiri et al. showed that *P. gingivalis* can inhibit apoptosis in gingival epithelial cells by upregulation of the anti-apoptotic molecule Bcl-2, whereas Bax levels were transiently elevated and then declined after 24 h stimulation [63]. *P. gingivalis* can also inhibit gingival epithelial cell apoptosis induced by ATP ligation of purinergic receptor P2X7, which plays a critical role in promoting cell growth, neovascularization, metastasis, and secretion of inflammatory cytokines. This bacterium has shown the ability to secrete an anti-apoptotic enzyme nucleoside diphosphate kinase (NDK), which cleaves ATP and prevents activation of the proapoptotic P2X7 receptor, therefore modulating ATP/P2X7-signaling [64]. Secretion of the NDK *P. gingivalis* can additionally modulate ATP-induced cytosolic and mitochondrial reactive oxygen species (ROS), as well as antioxidant glutathione response generated through P2X7/NADPH-oxidase interactome [65]. ROS can serve as a key mediator in the activation of transcription factors associated with inflammation and cancer development [66]. Moreover, *P. gingivalis* produces cysteine proteinases named gingipains, which can cleave the MMP-9 pro-enzyme, changing it into its mature active form. This process is NF-κB-dependent. Activation of MMP-9 by gingipains causes degradation of basement membrane structure, which promotes carcinoma cell migration and invasion [67]. Interactions between *Porphyromonas gingivalis* and epithelial cells that can affect development of oncogenic phenotype are presented in Figure 2.

**Figure 3:**
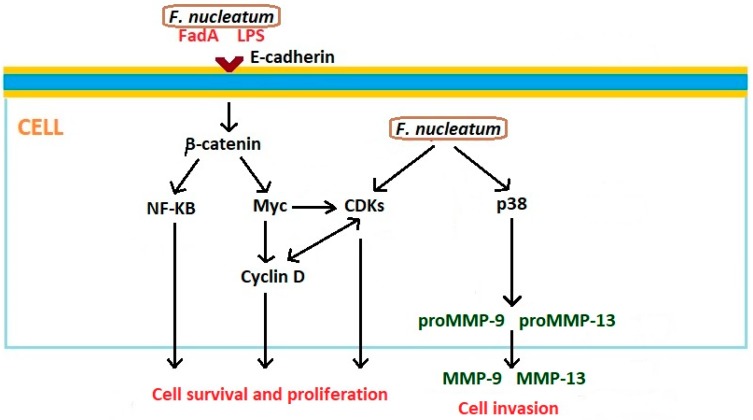
Interactions between *Fusobacterium nucleatum* and epithelial cells that can affect development of oncogenic phenotype. Based on Gholizadeh et al. [58] and Whitmore and Lamont [61]. CDK: cyclin-dependent kinase; FadA: fusobacterial adhesin/invasin; LPS: Lipopolisaccharide; MMP: metalloproteinase; NF-kB: nuclear factor kappa B; p38: Mitogen-activated protein kinase p38.

Examples of *Fusobacterium nucleatum* LPS-activated inflammatory cytokines are include IL-1β, IL-6, and TNF-α. The chronic inflammatory process leads to the loss of periodontal attachment and tissue damage [68]. *F. nucleatum* infection modulates several antiapoptotic pathways. Bacteria induce NF-kB signaling as a consequence of Toll-like receptor (TLR) activation [69]. Of importance in the direct relationship between *F. nucleatum* and cancer is the fusobacterial adhesin/invasin FadA, which binds to E-cadherin on carcinoma cells and activates β-catenin signaling. This pathway results in enhanced transcriptional activity of Wnt, activation of pro-inflammatory cytokines, oncogenes, and stimulation of cancer cells proliferation [70]. FadA is a key virulence factor of *F. nucleatum* and alters macrophage infiltration and methylation of the cyclin-dependent kinase inhibitor 2A (CDKN2A) promoter in cancer lesions [71]. *F. nucleatum* may also activate β-catenin signaling through its LPS. In this process, the enhancement of the expression of β-catenin, and oncogenes C-myc and cyclin D1, is observed [72,73]. Additionally, *F. nucleatum* activates p38, resulting in the secretion of MMP-9 and MMP-13, which play very important roles in the invasion of cancer cells and metastasis [74]. Interactions between *Fusobacterium nucleatum* and epithelial cells that can affect the development of oncogenic phenotype are presented in Figure 3.

### 3.3. Cancerogenic Substances

We have little knowledge of cancerogenic substances produced by oral bacteria. Substances that may have a carcinogenic effect include the following: reactive oxygen species (ROS) and reactive nitrogen species (RNS), volatile sulfur compounds (VSC), and organic acids. The metabolization of alcohol to acetaldehyde by micro-organisms also plays an important role in the development of cancer.

During an inflammatory response, under the influence of TNF-α, IL-6, and TGF-β, epithelial and immune cells trigger reactive oxygen species (ROS) and reactive nitrogen species (RNS) [75,76]. Production of ROS and RNS occurs through induction of NADPH oxidase and nitric oxide synthase (NOS), respectively. NADPH oxidase catalyzes the superoxide anion (O_2_^−^∙) leading to superoxide dismutase-(SOD^−^)-mediated hydrogen peroxide (H_2_O_2_) production. Simultaneously, NOS generates nitric oxide (NO), which can be converted into nitrogen dioxide (NO_2_), peroxynitrite (ONOO^−^), and dinitrogen trioxide (N_2_O_3_) [77]. Some species in the oral cavity involved in this process produce hydrogen peroxide (H_2_O_2_). Known peroxigenic oral bacteria include: *Streptococcus oralis*, *S. mitis*, *S. sanguinis*, *S. gordonii*, *S. oligofermentans* [78], *Lactobacillus fermentum*, *L. jensenii*, *L. acidophilus*, *L. minutus*, and *Bifidobacterium adolescentis* [79]. Increased expression of NADPH oxidase, nitric oxide synthase, and their reactive oxygen and nitrogen species have been identified in various cancers. These findings support the connection of free radicals with chronic inflammation and their role in cancer development and malignant progression [80,81].

Some oral bacteria (e.g., *Porphyromonas gingivalis*, *Prevotella intermedia*, *Aggregatibacter actinomycetemcomitans*, and *Fusobacterium nucleatum*) produce volatile sulfur compounds (VSCs), such as hydrogen sulfide (H_2_S), methyl mercaptan (CH_3_SH), dimethyl sulfide ((CH_3_)_2_S), and dimethyl disulfide (CH_3_SSCH_3_). H_2_S occurs in the highest concentration in the air inside the mouth, while in the gingival pockets the dominant compound is CH_3_SH [82,83]. Even at low concentrations, VSCs are toxic to tissues and play a role in the pathogenesis of periodontitis and in the development of chronic inflammation [84]. H_2_S is a known genotoxic agent and may lead to genomic instability or cumulative mutations [85]. Increased expression of various H_2_S-producing enzymes has been observed in cancer cells, particularly in cancers of the colon and ovaries. Overexpression of cystathionine-β-synthase causes the production of increased amounts of H_2_S, which affect tumor growth and spread by activation of proliferation, migration, and invasive signaling pathways, and enhance tumor angiogenesis [86]. Recently, H_2_S has been found to have dichotomous effects (stimulatory and inhibitory) on several gastrointestinal processes such as inflammation, cancer, and apoptosis [87].

Some oral bacteria belonging to genera *Lactobacillus*, *Lactococcus*, *Bifidobacterium*, *Streptococcus*, *Leuconostoc*, and *Pediococcus* produce lactic acid [88]. Hooper et al. reported that most taxa isolated from within the tumor tissue of oral squamous cell carcinoma represent saccharolytic and aciduric species, mainly streptococci [89]. These microorganisms are acidogenic and aciduric, and by producing lactic acid have an influence on lowering the pH in the local environment [90]. Some species are capable of producing more acids (e.g., aciduric *Peptostreptococcus stomatis* produces acetic, butyric, isobutyric, isovaleric, and isocaproic acids) [91]. Production of such acids may contribute to the acidic and hypoxic microenvironment of tumors, thereby increasing metastatic efficiency [92,93].

Oral micro-organisms are capable of metabolizing alcohol to acetaldehyde, which is indisputably carcinogenic. Several oral microbial species such as streptococci *S. gordonii*, *S. mitis*, *S. oralis*, *S. salivarius*, *S. sanguinis* [94], and *Candida* yeasts possess the enzyme alcohol dehydrogenase (ADH), which metabolizes alcohol to acetaldehyde [95]. ADH-containing micro-organisms present a risk for carcinogenic acetaldehyde production, with subsequent potential for the development of oral cancer [96,97]. Muto et al. showed that the genus *Neisseria* had extremely high ADH activity and produced significant amounts of acetaldehyde in vitro. *Neisseria*’s ability to produce acetaldehyde was more than 100-fold higher than that produced by *Streptococcus* sp., *Stomatococcus* sp., or *Moraxella* sp. The authors suggested that *Neisseria* can be a regional source of carcinogenic acetaldehyde and may thus play an essential role in alcohol-related carcinogenesis in humans [98].

## 4. Conclusions

Bacteria of the oral cavity play an important role in the development of oral, colorectal, and pancreatic cancers. The most well-confirmed is the carcinogenic effect of oral periopathogens: *Fusobacterium nucleatum* and *Porphyromonas gingivalis*. Others playing an essential role in cancerogenesis seem to be *Streptococcus* sp., *Peptostreptococcus* sp., *Prevotella* sp., and *Capnocytophaga gingivalis*. Bacteria can have an oncogenic effect on human cells in three ways: leading to chronic inflammation, acting as an antiapoptotic, and producing carcinogenic substances. However, further research is needed to clearly define specific oral bacteria as carcinogens.

## Figures and Tables

**Table 1:** Oral bacteria as biomarkers of specific cancer types.

Cancer Localization	Oral Bacteria as Biomarkers	Main Findings	Reference
Oral squamous cell carcinoma (OSCC)	*Streptococcus anginosus*	*S. anginosus* infection is more common in OSCC	[7]
Oral squamous cell carcinoma (OSCC)	*Capnocytophaga gingivalis*, *Prevotella melaninogenica*, *Streptococcus mitis*	Levels of mentioned bacteria were elevated in the saliva of patients with OSCC	[8]
Oral squamous cell carcinoma (OSCC)	*Bacillus*, *Enterococcus*, *Parvimonas*, *Peptostreptococcus*, *Slackia*	Significant differences between epithelial precursor lesion and cancer patients in presented five bacterial genera	[13]
Oral squamous cell carcinoma (OSCC)	*Streptococcus* sp. 058, *S. salivarius*, *S. gordonii*, *S. parasanguinis, Peptostreptococcus stomatis*, *Gemella haemolysans*, *G. morbillorum*, *Johnsonella ignava*	Presented bacteria were highly associated with OSCC tumor sites	[10]
Oral squamous cell carcinoma (OSCC)	*Capnocytophaga gingivalis*, *Prevotella melaninogenica*, *Streptococcus mitis*, *Porphyromonas gingivalis*	The high salivary counts of studied bacteria may be diagnostic indicators of oral squamous cell carcinoma	[12]
Gingival squamous cell carcinoma	*Porphyromonas gingivalis*	*P. gingivalis* was abundantly present in malignant oral epithelium	[9]
Oral mucosal cancer	*Streptococcus intermedius*, *S. constellatus*, *S. oralis*, *S. mitis*, *S. sanguis*, *S. salivarius, Peptostreptococcus* sp.	Bacteria were the most common isolates from cervical lymph nodes in patients with oral cancer	[24]
Head and neck squamous cell carcinoma (HNSCC)	*Streptococcus* sp. and *Lactobacillus* sp.	HNSCC saliva samples were associated with increased amounts of *Streptococcus* and *Lactobacillus* and a decrease in *Haemophilus*, *Neisseria*, *Gemella*, and *Aggregatibacter*	[30]
Head and neck squamous cell carcinoma (HNSCC)	*Streptococcus anginosus*	*S. anginosus* infection is implicated in the carcinogenesis of HNSCC	[22]
Keratinizing squamous cell carcinoma	*Veillonella* sp., *Fusobacterium* sp., *Prevotella* sp., *Porphyromonas* sp., *Actinomyces* sp., *Clostridium* sp., *Haemophilus* sp., *Streptococcus* sp., and Enterobacteriaceae	Higher numbers of presented bacteria in keratinizing squamous cell carcinoma	[14]
Orodigestive cancer	*Porphyromonas gingivalis*	*P. gingivalis* is a biomarker for microbe-associated risk of death due to orodigestive cancer	[31]
Esophageal cancer	*Streptococcus anginosus*, *S. mitis*, *Treponema denticola*	Studied bacteria could have a significant role in the carcinogenic process by causing inflammation and by promoting the carcinogenesis	[23]
Esophageal adenocarcinoma and esophageal squamous cell carcinoma	*Porphyromonas gingivalis*, *Tannerella forsythia*	The abundance of *P. gingivalis* is trended with higher risk of esophageal squamous cell carcinoma, and *T. forsythia* is associated with higher risk of esophageal adenocarcinoma	[32]
Colorectal cancer (CRC)	*Fusobacterium* sp., *Porphyromonas* sp.	Increased carriage of presented bacteria was found in patients with CRC; lower abundance of *Clostridium* sp. was simultaneously observed	[16]
Colorectal cancer (CRC)	*Fusobacterium* sp.	*Fusobacterium* enrichment is associated with specific molecular subsets of colorectal cancers	[26]
Colorectal cancer (CRC)	*Fusobacterium* sp.	*Fusobacterium* sp. are enriched in human colonic adenomas. *F. nucleatum* increases tumor multiplicity and can promote tumor progression	[17]
Colorectal cancer (CRC)	*Fusobacterium nucleatum*	Patients with low *F. nucleatum* levels had a significantly longer overall survival time than patients with moderate and high levels of the bacterium	[19]
Colorectal cancer (CRC)	*Fusobacterium* sp.	Overabundance of *Fusobacterium* in tumor has positive association with lymph node metastasis	[15]
Colorectal cancer (CRC)	*Fusobacterium* sp.	*Fusobacterium* sequences were enriched in CRC	[25]
Colorectal cancer (CRC)	*Fusobacterium* sp., *Lactococcus* sp.	Presented bacteria exhibited a higher abundance in cancerous tissues, while *Pseudomonas* and *Escherichia*-*Shigella* were reduced	[33]
Pancreatic cancer	*Porphyromonas gingivalis*	Individuals with high levels of antibodies against *P. gingivalis* had a higher risk of pancreatic cancer	[18]
Pancreatic cancer	*Porphyromonas gingivalis*, *Aggregatibacter actinomycetemcomitans*	Carriage of both pathogens was associated with higher risk of pancreatic cancer	[21]
Pancreatic cancer	*Fusobacterium* sp.	Level of *Fusobacterium* species in the tumor is associated with a worse prognosis of pancreatic cancer	[20]
Pancreatic cancer	*Streptococcus mitis, Neisseria elongata*	Bacteria can be used as biomarkers for distinguishing patients with pancreatic cancer from healthy subjects	[28]
Lung cancer	*Capnocytophaga* sp., *Veillonella* sp.	Levels of presented bacteria were significantly higher in the saliva from lung cancer patients	[29]
